# Antidepressant-like effects of Rg1 by mycn: promotion of neurogenesis in the hippocampus

**DOI:** 10.3389/fphar.2026.1734681

**Published:** 2026-03-10

**Authors:** Enze Shi, Yiwei Zhang, Yanyan Fan, Jiangfan Chen, Linfeng Xu, Jiao Chen

**Affiliations:** 1 Forensic Medicine, School of Basic Medical Science, Wenzhou Medical University, Wenzhou, China; 2 The State Key Laboratory of Optometry, Ophthalmology and Vision Science, Wenzhou Medical University, Wenzhou, China; 3 Electronic Materials Research Laboratory, Key Laboratory of the Ministry of Education & International Center for Dielectric Research, School of Electronic Science and Engineering, Xi’an Jiaotong University, Xi’an, China

**Keywords:** anxiety, depression, MYCN, neurogenesis, Rg1

## Abstract

Ginsenoside Rg1 has been shown to have antidepressant effects by increasing hippocampal neurogenesis, but the molecular mechanisms remain unclear. In this research, we showed that Rg1 has antidepressant-like effects by increasing neurogenesis in the hippocampus, and these effects are achieved through Mycn. The evidence shows that Rg1 has antidepressant like effects in the tail suspension test, sucrose preference test. Moreover, Rg1 has anxiolytic-like effects in the O-maze test. In addition, Rg1 increases Mycn mRNA expression by q-PCR test. Mycn overexpression in the DG of ventral hippocampus is stress resilient. Furthermore, Mycn inhibition makes the mice more susceptible to depression and Rg1 cannot rescue this effect. In conclusion, Ginsenoside Rg1 targets Mycn to increase hippocampal neurogenesis to alleviate depressive-like behaviors.

## Introduction

Depression is a psychiatric disorder marked by persistent sadness, loss of interest, and related mood disturbances (Kessler et al., 1994; Rice et al., 2019). From 2011 to 2020, the rate of depressive symptoms in adolescents rose from 24% to 37% (Shorey et al., 2022). But the mechanisms remain unknown. Current antidepressants are associated with significant side effects and fail to achieve full symptom resolution in nearly half of the patients, some individuals even experience relapse (McGrath et al., 2006). Therefore, there is a critical need for novel antidepressants with greater efficacy and improved safety profiles.

Ginseng, a cornerstone of traditional Chinese medicine for millennia, contains Ginsenoside Rg1 as one of its primary bioactive constituents. Research has demonstrated that Rg1 offers neuroprotective benefits, particularly against damage from ischaemia/reperfusion and the pathology of Alzheimer’s disease (Zhang et al., 2008; Wang and Du, 2009; Shi et al., 2010). Ginseng exerts antidepressant and anxiolytic effects by regulating the HPA axis and monoaminergic system (Fugh-Berman and Cott, 1999; Kim et al., 2003). Additionally, its active compounds have been found to provide neuroprotection and enhance neuronal survival. Rg1 stimulates the proliferation of hippocampal progenitor cells (Shen and Zhang, 2004). Ginsenoside treatment enhanced the survival neuronal plasticity and neurogenesis of dopaminergic cells (Radad et al., 2004). Studies have also shown that Ginsenoside can reduce hypoxic brain injury in rats (Ji et al., 2005) and protect against toxic injury in animal models of Parkinson’s disease (Van Kampen et al., 2003). Further research revealed that Ginsenoside Rg1 regulates synaptic plasticity and prevents synaptic deficit associated with depression. In CUS rat models, intraperitoneal injection of Rg1 alleviated depression-like behaviors and restored the expression of synaptic-related proteins (Han et al., 2024). Additional reports suggest that Rg1 exerts antidepressant effects by promoting hippocampal neurogenesis and activating the BDNF signaling pathway (Dang et al., 2009; Jiang et al., 2012). However, the specific molecular mechanism that Rg1 regulates neurogenesis remains unclear.

Mycn was initially identified as an oncogene amplified in human neuroblastoma, and its expression level is correlated with patient prognosis (Matthay et al., 2016; Floros et al., 2021). Mycn also plays a key role in both embryonic brain development and adult brain plasticity (Knoepfler et al., 2002; Li et al., 2022; Yang et al., 2022). Our previous research revealed that Mycn is expressed in specific brain regions, including the subventricular zone and the subgranular zone of the hippocampal dentate gyrus - the two primary sites for adult neurogenesis (Chen and Guan, 2022). Mycn is mainly expressed in the neural progenitor cells and immature neurons. Moreover, Mycn plays an important role in the proliferation, maturation and migration of the new-born neurons. Of note, there is no new-born neurons in the dentate gyrus with the Mycn conditional knockout from the neural progenitor cells (Chen and Guan, 2022). Taken together, these results indicate that Rg1 may regulate neurogenesis through Mycn.

In this study, we first confirmed the antidepressant effects of Rg1. We found that chronic Rg1 treatment can increase Mycn expression in the hippocampus. Then our results showed that Mycn inhibitor can induce depressive-like behaviors after mild stress and Mycn overexpression is stress resistant. Mycn inhibition can block the antidepressant effects of Rg1 treatment. Our research showed that Rg1 regulates neurogenesis through Mycn.

## Methods

### Animals

Male adult C57BL/6J mice (6–8 weeks of age, Shanghai JieSiJie Laboratory Animal Co., Ltd.) were purchased and used in accordance with protocols approved by the Institutional Ethics Committee for Animal Use in Research and Education at Wenzhou Medical University, China. The mice were housed three to five per cage in a controlled temperature (23 °C ± 1 °C) and 60% ± 2% humidity room. The room is under a 12 h light/dark cycle (light on from 8 a.m. to 8 p.m.) with *ad libitum* food and water.

### Chronic unpredictable stress (CUS) and chronic restraint stress (CRS)

Chronic unpredictable stress procedure involved exposing animals to a variety of different stressors, including food deprivation, water deprivation, soiled cage, crowding, isolation 24 h, 4 h immobilization, 1 h shaker stress (160 rpm), cage-tilt at 45° overnight, reversal of the light/dark cycle, ice water swim 5 °min, sleep deprivation overnight, 1 h cold stress at 4 °C. The mice received one or two of these stressors per day for a period of 4 weeks. We also used the chronic restraint stress (CRS) procedure to develop depressive mouse model (Qiao et al., 2016). The mice were restrained for 3 weeks, and the mice were restrained 3 hours per day for the first week, then the restraint time increased to 5 h per day for the last 2 weeks.

### Stereotactic injection

For viral injections, mice were anesthetized with Avertin (250 mg/kg, Sigma-Aldrich) and secured in a stereotaxic frame with ear bars. Using a 10 μl syringe fitted with a pulled glass capillary, 300 nl of either LV - Nestin - Mycn - EGFP - WPRE or LV- Nestin - EGFP – WPRE was bilaterally infused into the ventral dentate gyrus (vDG) of the hippocampus at the following coordinates: −3.6 mm AP, ±2.8 mm ML, and −3.2 mm DV. The virus was injected by a microsyringe pump controller (RWD Life Science). Following each injection, the needle was left in place for an additional 5 min to allow for viral diffusion to minimize backflow before being slowly withdrawn. The scalp incision was then closed with wound clips. Post-surgery, mice were placed on a heating pad to allow for recovery from anesthesia. All animals received post-operative carprofen (subcutaneous, 5 mg/kg) for analgesia and were monitored daily for 3 days. After the experiment, we checked all the animals that had the virus injection, only data from animals that had the virus expression in the vDG of the hippocampus were included in the analysis.

### Cannula infusion

A guide cannula (Kedou Brain-computer Technology Co., Ltd), consisting of an outer hollow catheter (0.5 mm outer diameter) and a protective cap with an inner core (0.25 mm outer diameter), was bilaterally implanted into the vDG of hippocampus (−3.6 mm AP, ±2.8 mm ML, and −3.2 mm DV) of C57BL/6J mice. Following implantation, a double dummy cannula with a 0.5 mm extension and a protective metal cap was inserted into the guide cannula. After a 1-week recovery period, mice received daily micro infusions of either the Mycn inhibitor MLN8237 (0.5 μg/μL) or vehicle (DMSO + PBS) for 21 consecutive days. For each infusion, 2 μL of the solution was delivered via a drug-delivery inner core connected to a microinjection pump. The injector was left in place for an additional 5 min post-infusion to facilitate local drug diffusion and minimize backflow. Only data from animals with histologically verified correct cannula placement, confirmed by HE-stain, were included in the analysis.

### Behavioral tests

All behavior tests were conducted between 10:00 a.m. and 5:00 p.m. inside a sound-attenuating booth. The performance of the animals was recorded and analyzed with the EthoVision XT system. The chamber was wiped down with 70% ethanol between each animal. The behavioral tests were performed by this order: open field test, sucrose preference test, O-maze, and tail suspension test, and the interval between each test was 24h. The experimenter who handled the behavioral tests was blind to the treatment or genotype.

### Open field test (OFT)

Animals were individually placed in the center of a chamber (40 × 40 × 40 cm). The chamber was in a soundproof environment with gentle light on the top. The movement of the animals was analyzed during 15 min.

### Tail suspension test (TST)

The mice were fixed upside down on a horizontal bar with the tape wrapped at approximately 1 cm from the end of the tail. The nose tip was about 30 cm above the ground. The performance of the mice was recorded for 6 min and the first min was used for the animals to habituate and only the last 5 min was analyzed to count the immobility time. When the animal was totally immobile without any movement of the body or limbs, that was defined as immobility, and the time was counted.

### Elevated O maze

The maze was constructed in an O-shape track, which was 10 cm wide, 105 cm in diameter, and elevated 72 cm from the floor. The maze was divided into four parts, two opposing open quadrants and two opposing closed quadrants, the closed quadrants are not physically closed from the other parts, each part was equal length. The two opposing open quadrants has 1 cm high curbs to prevent falls and the two opposing closed quadrants has walls 28 cm in height. The mouse was placed in the center of a closed quadrant for 5 min, during which the behavior of the mouse was recorded.

### Sucrose preference test (SPT)

Mice were single-housed and acclimated to 1% sucrose water for 48 h. On the test day, following 12 hours of food and water deprivation, animals were presented with two identical bottles – one containing water and the other 1% sucrose – for 2 hours. Sucrose preference was calculated as the ratio of sucrose solution consumed to total liquid (water and sucrose solution) intake. The positions of the two bottles were exchanged every 10 min to avoid side preference.

### RNA extraction and quantitative real-time PCR

Total RNA was extracted from mouse brain tissue using Trizol reagent (Invitrogen) according to the manufacturer’s protocol. Reverse transcription was performed with HiScript III RT Super Mix (Vazyme), with incubation at 42 °C for 2 min, followed by 37 °C for 15 min and 85 °C for 5 seconds. Real-time PCR was then carried out on a StepOne system using iTaq Universal SYBR Green Supermix (Bio-RAD). Q-PCR primers used were:

β-actin:

forward:5′-CCACACCCGCCACCAGTTCG-3′

reverse:5′-TACAGCCCGGGGAGCATCGT-3′

Mycn:

forward: 5′-AGC​GTT​CAA​CTA​GCA​GAC​CAT-3′

reverse:5′-CGAAAGGGCAGATTGTGTGG-3′

BDNF:

forward: 5′-GAC​AAG​GCA​ACT​TGG​CCT​AC-3′

reverse: 5′-CCT​GTC​ACA​CAC​GCT​CAG​CTC-3′

### Immunofluorescence staining

For immunofluorescence, mice were intracardially perfused with PBS followed by 4% paraformaldehyde (PFA, PH = 7.4) under anesthesia. Brains were harvested, post-fixed overnight in 4% PFA, and cryoprotected in 30% sucrose for 3 days. Coronal sections (20 μm) were cut on a cryostat (Leica CM1950) and processed as free-floating sections. After blocking (1h, RT) in PBS containing 0.3% Triton X-100 and 5% normal donkey serum, sections were incubated with primary antibodies overnight at 4 °C in antibody solution (0.3% Triton X-100, 1% bovine serum albumin in PBS). Following three PBS washes (10 min each), sections were incubated with secondary antibodies and DAPI for 2 h at RT, then washed again three times with PBS. The following antibodies were used: rabbit anti-doublecortin (DCX) (1:1000; Abcam); donkey anti-rabbit 488 (Invitrogen, 1:1000). All fluorescent image acquisition was performed with a Leica DM6B microscope and a Zeiss LSM880 NLO confocal microscope.

### EdU staining and counting

For EdU labeling, mice received an intraperitoneal injection of EdU (25 mg/kg; Invitrogen, C10337) 2 hours prior to tissue collection. Brain sections were incubated for 30 min with freshly prepared Click-iT reaction cocktail (containing 1 x Click-iT reaction buffer, CuSO4, Alexa Fluor azide, and 1 x reaction buffer additive), followed by three 10-min PBS washes. Sections were then incubated with primary antibody overnight, followed by second antibody for 1 h at room temperature, washed with PBS for 10 min, and counterstained with DAPI.

For the total EdU + cell number counting, we chose the whole DG of hippocampus to count the EdU + cell number. We cut the brain from the hippocampus and collected 20 μm cryosections of the hippocampus from three animals per group. We mounted, immuno-stained, and counted EdU + cells in every fourth section of the DG in the hippocampus. For the DCX/EdU double staining, we cut the DG of the ventral hippocampus from three animals per group, 20 μm cryosections were collected, and four slices per animal were used to do the DCX/EdU double staining.

### Statistical analysis

Data are expressed as mean ± SEM. Statistical analysis was performed using GraphPad Prism, version 8.0. Unpaired two-tailed Student’s t tests were used for single comparisons between two groups. Other data were analyzed using one-way analysis of variance (ANOVA) or two-way ANOVA followed by multiple comparisons test.

## Results

### Chronic Rg1 treatment reverses depressant-like behaviors and anxiety-like behaviors in chronic unpredictable stress (CUS) mice

**FIGURE 1:**
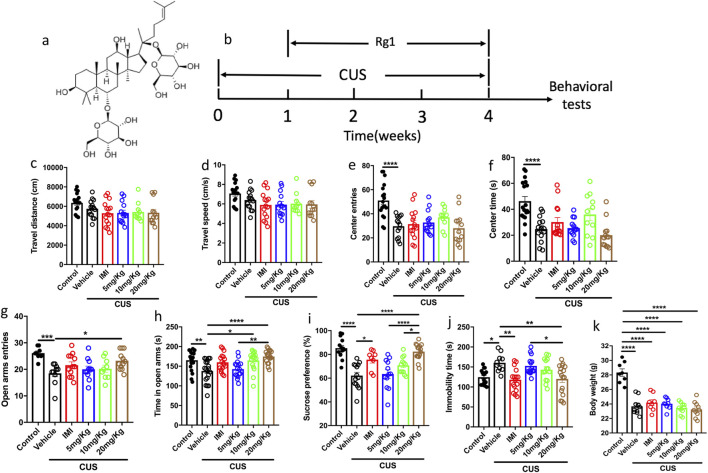
Rg1 has antidepressant-like effects and anti-anxious like effects in chronic unpredictable stress (CUS) mice. **(a)** The chemical structure of Ginsenoside Rg1. **(b)** The experimental design. Mice were exposed to CUS for 4 weeks and received saline (vehicle), imipramine (IMI, 15 mg/kg) or Rg1 (5, 10, 20 mg/kg) during the last 3 weeks. Behavioral tests were then conducted. **(c)** Rg1 had no effect on the total distance of the mice in the open field test. **(d)** Rg1 had no effect on the travel speed of the mice in the open field. **(e)** CUS led to less center entries in the vehicle group in the open field, while Rg1 had no effect on the center entries in the open field. **(f)** CUS led to less center time in the vehicle group in the open field, while Rg1 had no effect on the center time in the open field. **(g)** CUS led to less open arms entries in the vehicle group, and Rg1 reversed this trend in the O-maze test. **(h)** CUS led to less time spent in the open arms in the vehicle group, while Rg1 rescued it in a dose-dependent manner. **(i)** CUS led to less sucrose intake in the vehicle group, while Rg1 treatment produced robust antidepressant effects in the sucrose preference test in a dose dependent manner. **(j)** CUS led to more immobility time in the vehicle group, Rg1 decreased the immobility time in the tail suspension test (TST) in a dose-dependent manner. **(k)** CUS reduced the body weight of all the stressed groups. The data are expressed as means ± SEM (n≥8), *p < 0.05, **p < 0.01, ***p < 0.001, ****p < 0.0001, one-way ANOVA followed by multiple comparisons test.

To confirm the antidepressant effects of Rg1, we used chronic unpredictable stress (CUS), which is currently used as one of the most predictive animal models of depression (Chourbaji et al., 2005; Chen et al., 2016; Chen et al., 2019). In the present study, we used O-maze to test the effects of Rg1 on anxiety-like behaviors, also we used sucrose preference test and tail suspension test to examine the antidepressant effects of Rg1. Figure 1a shows the chemical structure of Rg1, and Figure 1b shows the experimental design. We first used the open field test to examine the free locomotor activity of the mice, the results showed that Rg1 had no significant effects on the travel distance and travel speed of the mice (Figures 1c,d). The vehicle/CUS group mice showed fewer center entries and spent less time than the control mice in the center after CUS (Figure 1e, control vs. vehicle/CUS, p < 0.0001; Figure 1f, control vs. vehicle/CUS, p < 0.0001), which indicated anxiety like behaviors after CUS, while Rg1 showed no effects in this anxiety-like behavioral model. CUS induced anxiety-like behaviors in vehicle/CUS group and Rg1 reversed these effects by increasing open arms entries and open arms spending time (Figure 1g, control vs. vehicle/CUS, p = 0.001; vehicle/CUS vs. 20 mg/kg, p = 0.049; Figure 1h, control vs. vehicle/CUS, p = 0.0054; vehicle/CUS vs. 10 mg/kg, p = 0.0118; vehicle/CUS vs. 20 mg/kg, p = 0.0001). Moreover, we also tested the depressive like behaviors of the mice, the results showed that CUS induced less sucrose solution consumption in the vehicle/CUS group, and Rg1 increased sucrose solution intake in a dose dependent manner, imipramine also increased sucrose solution consumption significantly (Figure 1i, control vs. vehicle/CUS, p < 0.0001; vehicle/CUS vs. IMI, p = 0.0167; vehicle/CUS vs. 20 mg/kg, p < 0.0001) in the sucrose preference test (SPT). Tail suspension test (TST) was also used to test the helpless behaviors of the mice, which indicated the depressive like behavior of the animals. CUS induced more immobility in the vehicle/CUS group in the TST, while Rg1 rescued this effect in a dose-dependent manner, imipramine also reversed the immobility time to the control level (Figure 1j, control vs. vehicle/CUS, p = 0.0167; vehicle/CUS vs. IMI, p = 0.0028; vehicle/CUS vs. 20 mg/kg, p = 0.0044). The body weight of all the stressed animals reduced significantly compared with that in the control group (Figure 1k, control vs. vehicle/CUS, p < 0.0001). Note that the Rg1 20 mg/kg dose has the best antidepressant effects in both SPT and TST. These findings suggest that Rg1 rescued depressant-like behaviors and anxiety-like behaviors in CUS mice.

### Chronic Rg1 treatment improves hippocampal neurogenesis in chronic unpredictable stress (CUS) mice

**FIGURE 2:**
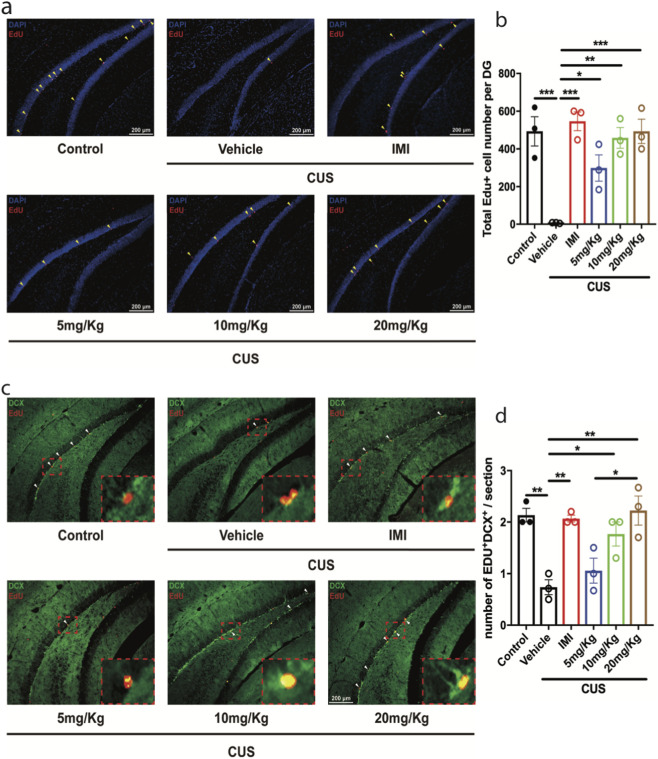
Rg1 reverses the decreased proliferation of hippocampal progenitor cells induced by CUS. **(a)** Representative images showed EdU staining in the DG. The scale bar is 200 μm. **(b)** The quantification of EdU positive cell number. The decrease of EdU positive cell number induced by CUS was reversed by Rg1 administration for 21 days (n = 3). **(c)** Representative images showed co-localization of EdU (red) and DCX (green) in the ventral DG. The scale bar is 200 μm. The enlarged images showed the co-localization of EdU (red) and DCX (green). **(d)** Quantification of EdU and DCX double positive cells in the ventral DG. Rg1 reversed the decrease of EdU and DCX double positive cells induced by CUS. The data are expressed as means ± SEM (n = 3), *p < 0.05, **p < 0.01, ***p < 0.001, one-way ANOVA followed by multiple comparisons test.

**FIGURE 3:**
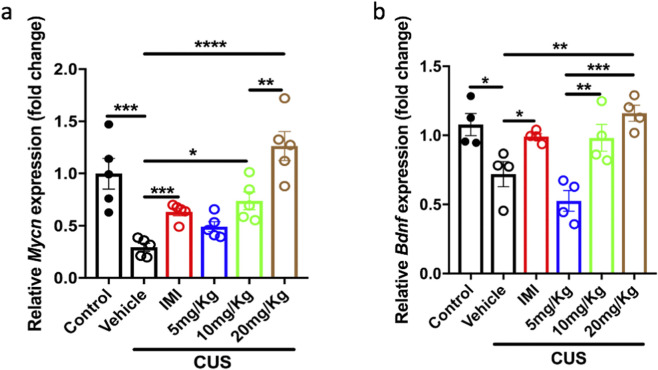
The decrease of Mycn and Bdnf mRNA level induced by CUS was reversed by Rg1 treatment. **(a)** Mycn mRNA level deduction was reversed by Rg1 treatment in a dose-dependent manner. **(b)** Bdnf mRNA level deduction was reversed by Rg1 treatment in a dose-dependent manner. The data are expressed as means ± SEM (n > 3), *p < 0.05, **p < 0.01, ***p < 0.001, ****p < 0.0001, one-way ANOVA followed by multiple comparisons test. Unpaired two-tailed Student’s t tests were used for single comparisons between two groups.

To confirm the improving effects of Rg1 treatment in the hippocampal neurogenesis, we used EdU to label the new-born cells in the dentate gyrus of the hippocampus and DCX as the marker of immature neurons. The results showed that CUS reduced EdU positive cell number almost to zero in the vehicle group, and both imipramine and Rg1 treatment increased total EdU number (Figures 2a,b control vs. vehicle/CUS, p = 0.0008; vehicle/CUS vs. IMI, p = 0.0003; vehicle/CUS vs. 5 mg/kg, p = 0.0369; vehicle/CUS vs. 10 mg/kg, p = 0.0015; vehicle/CUS vs. 20 mg/kg, p = 0.0008). And Rg1 increased the immature neuron, the EdU^+^DCX^+^ cell number, which was decreased by CUS (Figures 2c,d control vs. vehicle/CUS, p = 0.0033; vehicle/CUS vs. IMI, p = 0.0049; vehicle/CUS vs. 10 mg/kg, p = 0.0294; vehicle/CUS vs. 20 mg/kg, p = 0.002). These data indicated that chronic Rg1 treatment improves hippocampal neurogenesis. This data is consistent with Figure 1 that Rg1 has antidepressant effects in a dose-dependent manner. We further investigated the possible mechanisms underlying Rg1 induced neurogenic and antidepressant effects, we were interested in evaluating whether Rg1 influences neurogenesis through modifying Mycn and BDNF. We then examined the expression of Mycn and BDNF. Mycn is essential for neurogenesis (Chen and Guan, 2022), and BDNF is important for neuronal survival and neurogenesis in the brain (Sahay and Hen, 2007). Therefore, we measured BDNF and Mycn mRNA levels in the hippocampus after CUS. The Mycn and BDNF mRNA expression was expressed as a ratio of the expression of β-actin. The data showed that the average Mycn mRNA level was decreased in the hippocampus of mice exposed to CUS compared to the unstressed control group, while chronic Rg1 treatment increased the Mycn mRNA level in a dose dependent manner (Figure 3a control vs. vehicle/CUS, p = 0.0003; vehicle/CUS vs. 10 mg/kg, p = 0.0294; vehicle/CUS vs. 20 mg/kg, p < 0.0001; vehicle/CUS vs. IMI, p = 0.0002). Consistent with Mycn, we observed that the expression level of BDNF decreased after CUS, while Rg1 treatment increased BDNF level dose dependently (Figure 3b control vs. vehicle/CUS, p = 0.0318; vehicle/CUS vs. 20 mg/kg, p = 0.0063; vehicle/CUS vs. IMI, p = 0.0266). These data indicated that Rg1 treatment restored the neurogenesis impairment as well as Mycn and BDNF downregulation induced by stress.

### Mycn inhibition induces depressant-like behaviors and anxiety-like behaviors after mild stress

**FIGURE 4:**
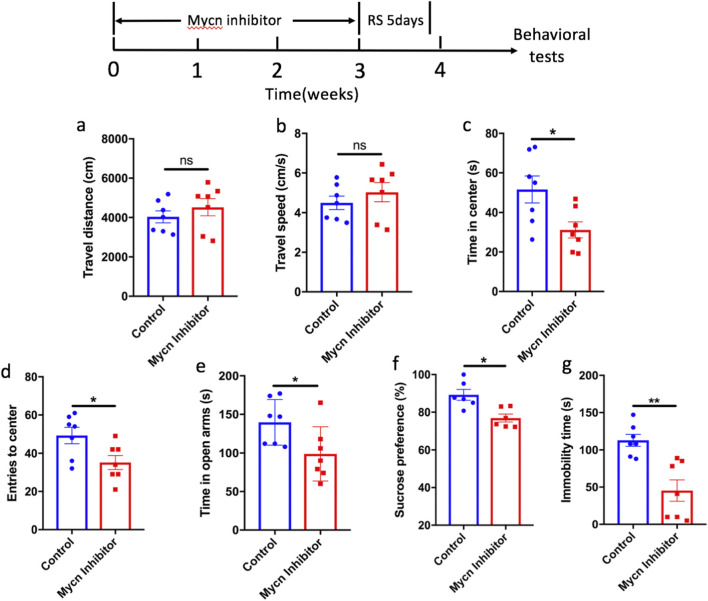
Mycn inhibition induces depressant-like behaviors and anxiety-like behaviors after 5 days of restraint stress. The first panel shows the experiment design. The Mycn inhibitor was delivered by cannula infusion for 3 weeks, then followed by 5 days of restraint stress. The depression and anxiety symptoms were assessed by behavioral tests, including open field test (OFT), O-maze and sucrose preference test (SPT). **(a)** Mycn inhibitor had no effect in the travel distance of the mice in the open field test. **(b)** Mycn inhibitor had no effect in the travel speed of the mice in the open field test. **(c)** Mycn inhibitor induced less time spent in the center compared with the mice in the control group in the open field test, which indicated anxiety-like behaviors. **(d)** Mycn inhibitor induced less entries to the center compared with the mice in the control group in the open field test, which indicated anxiety-like behaviors. **(e)** Mycn inhibitor mice spent less time than the control mice in the open arms in O-maze, which indicated anxiety-like behaviors. **(f)** Mycn inhibitor mice had less sucrose than the control mice in the sucrose preference test, which indicated depressant-like behaviors. **(g)** Mycn inhibitor mice had less immobility time than the control mice in the tail suspension test. The data are expressed as means ± SEM (n≥6), *p < 0.05, unpaired two-tailed Student’s t tests.

Next, we want to know whether inhibiting Mycn can induce depressant-like or anxiety-like behaviors. We used Mycn inhibitor MLN8237, which is believed to disrupt the Aurora-A/MYCN complex, thereby promoting the degradation of the MYCN protein mediated by Fbxw7 ubiquitin ligase (Brockmann et al., 2016), to inhibit the activity of Mycn. We delivered the inhibitor by cannula infusion, and 2 μL of MLN (0.5 μg/μL) was delivered to the ventral hippocampus for 3 weeks, then the mice were exposed to 5 days of restraint stress. The results showed that Mycn inhibitor had no significant effects in the travel distance (Figure 4a) and travel speed (Figure 4b) of the mice in the open field, but they spent less time in the center (Figure 4c, p = 0.0235) and had fewer entries (Figure 4d, p = 0.0271) to the center after Mycn inhibitor infusion compared with the control mice. Consistent with open field test, we also observed that the mice spent less time in the open arms in the O-maze (Figure 4e, p = 0.0363) after Mycn inhibitor infusion compared with the control mice. These data indicated anxiety-like behaviors of the mice induced by Mycn inhibitor. Furthermore, we also observed depressive-like behavior of the mice after Mycn inhibitor treatment supporting by data that Mycn inhibitor induced less sucrose preference compared with the control group (Figure 4f, p = 0.006). Of note, the mice did not show much immobility time than that in the control group after Mycn inhibition (Figure 4G, p = 0.0015) and they were restless. This data indicated that Mycn inhibition induced anxiety-like behavior in the tail suspension test. All these data demonstrated that Mycn inhibitor induces both depressant-like behaviors and anxiety-like behaviors after mild stress.

### Mycn overexpression is stress resilient

**FIGURE 5:**
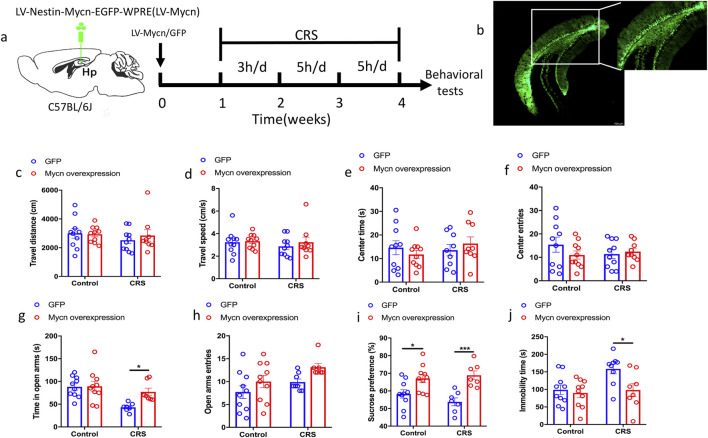
Mycn overexpression is stress resilient. **(a)** Experimental design. Mice were injected with LV-Nestin-Mycn -EGFP or LV-Nestin-EGFP to the ventral dentate gyrus (vDG), allowed to recovery for 1 week, and then subjected to chronic restraint stress (CRS) for 3 weeks 3 h of restraint per day for the first week, and 5 h of restraint per day for the last 2 weeks. The mice were assessed by open field test (OFT), O-maze, sucrose preference test (SPT), and tail suspension test (TST). **(b)** Representative image of virus injection to the vDG. Scale bar is 100 μm. **(c–f)** Mycn overexpression or CRS had no effect on the spontaneous locomotor activity of the mice in the OFT, including travel distance, travel speed, center time or center entries. **(g)** Mycn overexpression is anxiolytic. CRS induced less time spent in the open arms in the GFP mice, while mycn overexpression reversed this decrease, which indicated that mycn overexpression is anxiolytic. **(h)** Mycn overexpression had no significant effect on the open arms entries. **(i)** The sucrose consumption was significantly decreased by CRS and this effect was reversed by mycn overexpression in SPT. **(j)** The immobility time was significantly increased by CRS and this effect was reversed by mycn overexpression in TST. The data are expressed as means ± SEM (n≥7), *p < 0.05, ***p < 0.001, two-way ANOVA, followed by multiple comparisons test.

To test whether Mycn overexpression can be stress resilient, LV-Nestin-Mycn-EGFP-WPRE or LV-Nestin-EGFP-WPRE virus was injected to the ventral DG of hippocampus bilaterally to overexpress Mycn in this brain region. Chronic restraint stress for 3 weeks was used to develop the depression animal model (Figure 5a). For the first week, the mice were restrained 3 h per day, then the restraint time was increased to 5 h per day for the last 2 weeks.

**FIGURE 6:**
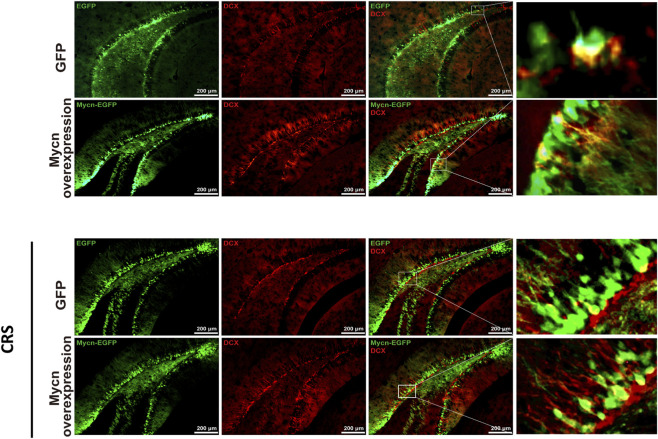
Mycn overexpression contributes to neurogenesis in the subgranular zone (SGZ) of DG in the ventral hippocampus. Immunostaining for GFP (green), Mycn-GFP (green) and DCX (red) in the SGZ of vDG in hippocampus. The enlarged images show the colocalization of DCX with GFP or Mycn-GFP.

Fluorescent images showed the mycn overexpression in the vDG of hippocampus after virus injection (Figure 5b). The results showed that the stress had no significant effects on the activity of the mice. There is no significant difference in the travel distance and travel speed after 3 weeks of restraint stress compared with the unstressed groups (Figure 5c, control: GFP vs. Mycn overexpression, p = 0.9844; CRS: GFP vs. Mycn overexpression, p = 0.7015; Figure 5d, control: GFP vs. Mycn overexpression, p = 0.9731; CRS: GFP vs. Mycn overexpression, p = 0.6868). In addition, the mice did not show any difference in center time and entries to the open field center (Figure 5e, control: GFP vs. Mycn overexpression, p = 0.6555; CRS: GFP vs. Mycn overexpression, p = 0.6946; Figure 5f, control: GFP vs. Mycn overexpression, p = 0.2925; CRS: GFP vs. Mycn overexpression, p = 0.9331). We further found that Mycn overexpression is anxiolytic supported by data that Mycn overexpression mice (Mycn-GFP group) spent more time in the open arms after stress compared with the GFP group (Figure 5g, control: GFP vs. Mycn overexpression, p = 0.9928; CRS: GFP vs. Mycn overexpression, p = 0.0329), while there is no significant difference in the entries to the open arms after Mycn overexpression compared with the GFP group, even though there is a trend that Mycn overexpression is likely to have more entries to the open arms (Figure 5h, control: GFP vs. Mycn overexpression, p = 0.2928; CRS: GFP vs. Mycn overexpression, p = 0.1652). We also found that Mycn overexpression is antidepressant by data that Mycn overexpression mice (Mycn-GFP group) had more sucrose solution compared with the GFP group both in the non-stressed and stressed groups (Figure 5i, control: GFP vs. Mycn overexpression, p = 0.0178; CRS: GFP vs. Mycn overexpression, p = 0.0007). Consistent with this result, we also observed that Mycn overexpression mice (Mycn-GFP group) showed less immobility time compared with the GFP group in the tail suspension test (Figure 5j, control: GFP vs. Mycn overexpression, p = 0.8769; CRS: GFP vs. Mycn overexpression, p = 0.0158). We also checked the DCX expression in the vDG after the Mycn overexpression. We found that some DCX colocalized with Mycn-GFP or GFP in the SGZ of DG (Figure 6), which indicated that they are immature neurons, and Mycn overexpression can stimulate DCX expression in this region, which suggested the neurogenesis promotion induced by Mycn. All these data indicated that Mycn overexpression reverses both depressive and anxiety-like behaviors induced by stress.

### Mycn is essential for the antidepressant effects of Rg1

**FIGURE 7:**
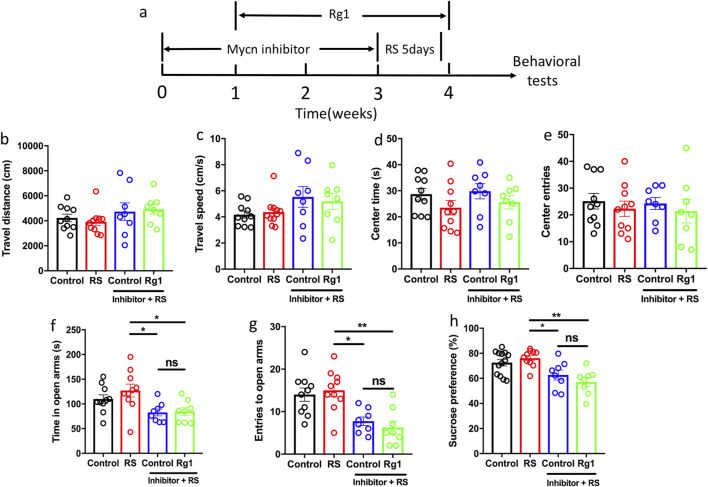
Mycn is essential for the anxiolytic and antidepressant like effects of Rg1. **(a)** Experimental design. The mice were administered Mycn inhibitor (20 mg/kg) every other day by intraperitoneal injection for 3 weeks, followed by 5 days of restraint stress. The mice were treated with Rg1 (20 mg/kg) for the last 3 weeks, and then they were subjected to behavioral tests, including OFT, O-maze, SPT. RS: restraint stress, 5 days. **(b,c)** Mycn inhibitor, Rg1 treatment or RS had no significant effect on the locomotor activity of mice in the OFT, including travel distance, travel speed. **(d,e)** Mycn inhibitor, Rg1 treatment or RS had no significant effect on the center time or center entries. **(f)** Time in open arms was decreased by Mycn inhibitor treatment plus RS, while Rg1 treatment had no significant effect on this behavior. **(g)** Entries to open arms was decreased by Mycn inhibitor treatment plus RS, while Rg1 treatment had no significant effect on this behavior. **(h)** Mycn inhibitor treatment plus RS mice showed less sucrose intake, while Rg1 could not rescue this behavior. The data are expressed as means ± SEM (n≥8), *p < 0.05, **p < 0.01, one-way ANOVA followed multiple comparisons test.

To investigate whether Mycn is essential for the antidepressant effects of Rg1, we used Mycn inhibitor (20 mg/kg) every other day by intraperitoneal injection for 3 weeks, and mild stress to induce depressive-like behaviors (Zhang et al., 2021), while Rg1 was administered to treat the depressive-like behaviors at the same time (Figure 7a). The results showed that Mycn inhibitor and Rg1 treatment had no significant effects on the locomotor activity of the mice in the open field test (Figures 7b,c). We also did not observe any significant difference in the center time and center entries after Mycn inhibitor and Rg1 treatment (Figures 7d,e). Furthermore, we found that Mycn inhibition induced anxiety-like behaviors after 5 days restraint stress (RS) demonstrated by data that the mice from Mycn inhibitor plus RS group spent less time in the open arms and had less entries to the open arms compared with RS group in the O-maze test, while Rg1 treatment could not reverse this effect (Figure 7f, RS vs. (inhibitor + RS), p = 0.0178; RS vs. (inhibitor + RS + Rg1), p = 0.0246; (inhibitor + RS) vs. (inhibitor + RS + Rg1), p = 0.9993; Figure 7g, RS vs. (inhibitor + RS), p = 0.0078; RS vs. (inhibitor + RS + Rg1), p = 0.0011; (inhibitor + RS) vs. (inhibitor + RS + Rg1), p = 0.9035). This data indicated that Mycn is essential for the anxiolytic effects of Rg1. Consistent with this result, we also found that Mycn inhibition induced depressive-like behavior after 5 days RS. The data showed that the mice from Mycn inhibitor plus RS group had less sucrose solution consumption compared with the RS group, while Rg1 could not rescue this anhedonia behavior induced by Mycn inhibition (Figure 7h, RS vs. (inhibitor + RS), p = 0.021; RS vs. (inhibitor + RS + Rg1), p = 0.0007; (inhibitor + RS) vs. (inhibitor + RS + Rg1), p = 0.6378). This data revealed that Mycn is essential for the antidepressant-like effect of Rg1. All in all, these results suggest that Mycn is necessary for the anxiolytic and antidepressant-like effects of Rg1.

## Discussion

In the present study, we have confirmed that Rg1 had antidepressant and anxiolytic effects in rodent models of depression. This antidepressant effect was mediated by neurogenesis through Mycn. The results demonstrate that animals exposed to CUS exhibit behavioral deficits in tests measuring anhedonia and anxiety, as well as hippocampal neurogenesis decrease and reductions in Mycn and BDNF mRNA levels in the hippocampus. The behavioral and cellular alterations induced by CUS were reversed both by chronic treatment with Rg1 or imipramine. Specifically, the behavioral changes induced by CUS is related to Mycn. The results demonstrate that pharmacological inhibiting Mycn activity in the hippocampus is sufficient to induce depressive-like and anxiety-like behaviors under mild stress similar to those induced by CUS. And overexpression of Mycn is stress resilient in CRS induced depression model. Rg1 had no significant effects in the stress induced behavioral deficits after Mycn inhibitor treatment. Together, our data support that Mycn dysregulation in the hippocampus may contribute to the expression of the depressive-like and anxiety-like symptoms, and Rg1 targets Mycn to improve neurogenesis in the hippocampus, thus has beneficial effects on CUS induced depression and anxiety.

Even though Rg1was shown to increase neurogenesis, thus has antidepressant effects, but the specific mechanisms that regulate neurogenesis is not clear. Our study provides direct evidence that Rg1 targets Mycn to improve neurogenesis and leads to antidepressant effects.

The CUS model is a widely used animal model of depression, valued for its high predictive, face, and construct validity (Willner, 2005). It is designed to mimic the stressful life events that contribute to human depression, producing behavioral and neuroendocrine alterations similar to those seen in patients (Willner, 1997). In our study, CUS exposure successfully reduced sucrose preference and increased immobility time in the tail suspension test, reflecting anhedonia and helpless behavior in animals (Willner et al., 1992). We also showed that the same procedure induces anxiety, demonstrated by decreased entries and time spending in the open arms in the O-maze. The behavioral alterations induced by CUS were reversed by chronic treatment with Rg1 and antidepressant imipramine as expected, consistent with the previous researches (Jiang et al., 2012; Yang et al., 2023).

Chronic stress induces atrophy of CA3 pyramidal neurons and suppresses hippocampal neurogenesis. These effects are thought to result from prolonged elevation of corticosteroids and excitatory amino acids (Sapolsky, 1996; Lupien et al., 1999), and all those changes can be reversed by antidepressant treatment (Watanabe et al., 1992), as well as rescued by chronic Rg1 treatment (Jiang et al., 2012). In this study, Rg1 administration reversed the decreased neurogenesis caused by CUS, which is consistent with previous studies. More interestingly, our results also showed that Rg1 upregulated the expression of Mycn mRNA as well as BDNF mRNA in the hippocampus of stressed mice. My previous study showed that Mycn plays an important role in adult neurogenesis (Chen and Guan, 2022) and neurogenesis is necessary for the activity of antidepressants in depressed patients and depressed animals (Smith et al., 1995; Dranovsky and Hen, 2006). Ablating hippocampal neurogenesis abolishes the effects of antidepressants in behavioral paradigms modeling depression- and anxiety-like responses in mice (Santarelli et al., 2003). In this study, the authors used X-irradiation of the SGZ to prevent neurogenesis. And they used Novelty-suppressed feeding and grooming test to evaluate the depressive like behaviors. The data showed that the antidepressants fluoxetine or imipramine could not rescue the depressive like behaviors induced by CUS. However, some researchers have the contrary results (Jedynak et al., 2014), they found that the antidepressant fluoxetine could reverse the depressive like behaviors in cyclin D2 knockout mice after unpredictable chronic mild stress, even though there is no neurogenesis in the cyclin D2 knockout mice. They used tail suspension test and forced swim test to measure the depressive like behaviors of the animals. How to explain the discrepancy? It is probably that they use different behavior models to measure the depressive behaviors, and the depression mechanism is complex, different behavior models may involve different mechanisms, only disruptions in neurogenesis may show some depressive symptoms but not all the symptoms.

Mounting evidence showed that stress and antidepressant treatment, especially Rg1 treatment, regulates neurogenesis in animals (Santarelli et al., 2003; Banasr et al., 2004). Increasing Mycn might be one of the cellular and molecular mechanisms by which this neurogenesis regulation takes place. In this study, Rg1 reversed the CUS induced decrease in the mRNA level of hippocampal Mycn and BDNF, as well as the decrease in the number of new-born neurons. In addition, BDNF-TrkB is required for the behavioral effects of antidepressants (Saarelainen et al., 2003; Monteggia et al., 2004). It is reported that deletion of BDNF from dentate gyrus cells of the mice blocks the effects of antidepressants in behavioral paradigms (Adachi et al., 2008). Further, deletion of TrkB from the progenitor cells of dentate granule neurons, rather than the mature granule neurons, inhibits the effects of antidepressants on the forced swim test as well as on induced neurogenesis (Li et al., 2008), these researches suggest that TrkB in the progenitor cells of the dentate gyrus is the target of BDNF. It is interesting to note that Mycn is mainly expressed in the progenitor cells of dentate gyrus, which is reported by our previous research (Chen and Guan, 2022). It is worthy of research to figure out whether if BDNF-TrkB signaling is down stream of Mycn to regulate the neurogenesis in the dentate gyrus. It is also interesting to investigate the upstream signaling molecules of Mycn. Previous researches showed that the antidepressant effects of Rg1 is to restore the hyperfunction of HPA axis, modulate GR expression and improve neurogenesis (Yang et al., 2023). GR is reported to regulate neurogenesis by data that GR heterozygous mice (GR (+/−)) showed reduced neurogenesis and depressive-like behaviors (Kronenberg et al., 2009). This indicated that Rg1 regulates neurogenesis may through GR. Further research showed that GR is mainly expressed in neural precursor cells and 50% of the radial glia-like type-1 and type-2a progenitor cells expressed GR (Garcia et al., 2004). It is possible that the antidepressant effects of Rg1 by neurogenesis promotion may involve GR and Mycn, GR maybe the upstream of Mycn, which needs further research.

We then examined whether inhibiting Mycn can induce depressive or anxiety-like behaviors. Our previous study showed that Mycn is expressed in specific brain regions including subgranular zone of dentate gyrus and subventricular zone, the two regions that neurogenesis happens. And Mycn has an important role in neurogenesis regulation (Chen and Guan, 2022). As expected, when we inhibit Mycn activity in the dentate gyrus of ventral hippocampus, the mice showed depressive and anxiety-like behaviors after subthreshold restraint stress when compared with the control group. This indicated that Mycn dysfunction is essential for stress induced depressive and anxiety-like behaviors. Mycn inhibitor might inhibit the expansion of neural progenitor cell populations during the neurogenesis (Knoepfler et al., 2002), thus reduce the number of new-born neurons. Interestingly, Mycn inhibition under mild stress induced depressive-like behaviors in SPT and anxiety-like behaviors in the O-maze, but not the TST, since we found that the Mycn inhibition induced more movements rather than more immobility in this test. We also found this phenotype in our Mycn conditional knock out (KO) mice, the mice were restless in the TST after the Mycn conditional KO in the neuronal progenitor cells (unpublished data). It is probably that the TST paradigm may involve different mechanism, as we mentioned above that different behavioral models may involve different mechanisms, Mycn inhibition can induce depressive-like behaviors in some behavioral models but not all.

What is more, consistent with this result, we also observed that the mice were stress resilient when Mycn was overexpressed in the dentate gyrus of ventral hippocampus. This stress resilient effect might be caused by neurogenesis enhancement due to Mycn overexpression. Mycn overexpression can promote the progenitor proliferation and neuronal differentiation (Mobley et al., 2015), thus buffer the stress induced depressive and anxiety-like behaviors. This data indicated that Mycn is one of the molecular mechanisms that neurogenesis regulation happens and at the same time Mycn is also one of the molecules that connects neurogenesis and affective behaviors.

In addition, hippocampal neurogenesis is essential for the behavioral effects of antidepressants in the chronic unpredictable stress model, as its ablation renders these drugs ineffective in mice displaying depressive-like and anxiety-like behaviors (Dranovsky and Hen, 2006). In this experiment we also tested whether Rg1 has antidepressant or anxiolytic effects when we inhibit the Mycn activity. Consistent with the previous reports, our results showed that Rg1 has no significant effects for the depressive and anxiety-like behaviors when the mice were treated with Mycn inhibitor after mild stress. This result indicated that Mycn is necessary for the Rg1 to exert the antidepressant and anxiolytic effects in depressive mouse model.

In summary, the present study shows that Rg1 exhibited antidepressant-like and anxiolytic effects in animal models of depression, which appeared to be mediated by Mycn. This study showed the cellular and molecular mechanism that through which the Rg1 regulates neurogenesis. This newly discovered target of Rg1 provides a new insight to understand the pharmacological effects of Rg1. More importantly, Mycn can be the new target for treating depression and anxiety.

Limitations: In this study we mainly used male animals as our study subjects, as we know, women are vulnerable to depression and there are gender differences in depression, the etiology is unknown. But hormones such as estrogens and glucocorticoids are known to influence depression. Women exhibit greater hypothalamic-pituitary-adrenal (HPA) axis activation in response to stress, and this dysregulation is most pronounced in menopausal women with diminished estrogen levels (Young and Korszun, 2010). In order to exclude the effects of hormones, male animals were used in this study, but it is still worthy to further study sex differences underlying depression.

For the design to test the antidepressant effects of Rg1, we did not arrange the control group receiving Rg1 to exclude the confounding effect of Rg1, due to the fact that other researches had tested that Rg1 has no side effects for the control animals (Jiang et al., 2012; Han et al., 2024).

## Data Availability

The raw data supporting the conclusions of this article will be made available by the authors, without undue reservation.
